# High-Performance Method of Recovery of Metals from EAF Dust—Processing without Solid Waste

**DOI:** 10.3390/ma14206061

**Published:** 2021-10-14

**Authors:** Stanisław Małecki, Krzysztof Gargul, Marek Warzecha, Grzegorz Stradomski, Artur Hutny, Mateusz Madej, Michał Dobrzyński, Ryszard Prajsnar, Grzegorz Krawiec

**Affiliations:** 1 Faculty of Non-Ferrous Metals, AGH University of Science and Technology, al. Mickiewicza 30, 30-059 Krakow, Poland; krzygar@agh.edu.pl; 2 Faculty of Production Engineering and Materials Technology, Czestochowa University of Technology, al. Armii Krajowej 19, 42-201 Czestochowa, Poland; marek.warzecha@pcz.pl (M.W.); grzegorz.stradomski@pcz.pl (G.S.); artur.hutny@pcz.pl (A.H.); 3 Przedsiębiorstwo Produkcyjno-Handlowo-Usługowe STILMAR, ul. Hallera 6-D, 42-200 Czestochowa, Poland; mateusz.madej@stilmar.pl (M.M.); michal.dobrzynski@stilmar.pl (M.D.); 4 Department of Metallurgy, Łukasiewicz Research Network—Institute of Non-Ferrous Metals, ul. Sowińskiego 5, 44-100 Gliwice, Poland; ryszard.prajsnar@imn.lukasiewicz.gov.pl (R.P.); grzegorz.krawiec@imn.lukasiewicz.gov.pl (G.K.)

**Keywords:** reduction, zinc recycling, iron recycling, steel, slag

## Abstract

A highly effective method of the processing of steelmaking dust in an arc-resistant furnace has been presented. The aim of the research was to investigate the possibility of processing steelmaking dust in terms of waste minimization and selective recovery of valuable components. For this purpose, an electric arc resistance furnace was used. Granulated steelmaking dust with reducer (coal dust) was the input material. The products of the process are zinc oxide, iron alloy and slag, with properties meeting high ecological requirements. The technology does not generate solid waste. Zinc recovery is over 99% and iron recovery over 98%. The content of heavy metals (Zn + Pb + Cu) in glassy slag is below 0.2%, which ensures very low leachability.

## 1. Introduction

Steel production in electric arc furnaces (EAF) generates large amounts of dust which, due to the content of heavy metals, should be considered a hazardous waste. The dust constitutes between 1 and 2% of the batch intended for melting [1,2]. As per [2,3] average annual steel production is 1600 million tons, of which 33% is produced in an electric arc furnace (EAF). This means that an annual output of 5 to 10 million tons EAF dust is generated.

The main ingredients of dust are zinc oxide (ZnO) and zinc ferrite (franklinite, ZnFe_2_O_4_) with the content of other iron oxides (Fe_x_O_y_). The content of zinc in dusts is up to 35%, while the iron content is 25–50%. The other ingredients of EAF dust are lead oxide, zinc chloride, heavy metal salts (e.g., PbCl_2_) and alkaline metal chlorides [1,4,5,6,7].

Currently, the most popular technology for recycling dust from electric arc furnaces containing zinc and iron is the Waelz Process, where dust from EAFs is placed in a rotary kiln with a load of coke [8,9,10]. This method treats 75% of the steel dust generated in EAF globally [2]. The reductor batch is treated at temperatures above 1200 °C. The diffusion of oxygen from the reaction atmosphere into the material bed is limited by air flow control. Coke and CO (from partial oxidation of carbon) reduce zinc oxide and zinc ferrite to zinc metal [8,11,12,13,14,15]. Zinc enters the gas phase and is oxidised in the gas stream to ZnO, which is collected on bag filters. This process produces slag in quantities of 600–800 kg/Mg of EAF dust, equivalent to over 2 million tons of “Waelz slag” in annual world production. Slag from the Waelz process is used mainly in construction and road building (as an aggregate); in the production of cement, concrete and bricks; as a basic material for sports fields and dykes; and as a drainage layer for landfill sites. However, it requires purification or binding of harmful components (lead, chromium, zinc, arsenic, and nickel), which, due to the nature of the slag phase (acidic or alkaline), may generate additional costs [16,17]. It should be added that the iron contained in the dust passes into the slag and is not recovered. Furthermore, these slags contain up to 2% zinc, which is lost.

Other processes like RHF, PRIMUS, OXYCUP, OXYFINES and Ausmelt [1,13,18,19,20] are less significant. These processes are intended for either zinc or iron recovery. There is virtually no process that comprehensively recovers both of the primary metals contained in EAF dust. An exception is the ESRF process [21], which is not currently in use.

Processes involving hydrometallurgical recovery of metals (mainly zinc) from steelmaking dust are not popular. Despite low operating costs of equipment and a more favourable energy balance, compared to pyrometallurgical processes, hydrometallurgical methods require great meticulousness and control of many parameters (concentration, pH of the solution) to maintain adequate efficiency of the zinc extraction process. This applies to both acidic (sulphuric acid) and alkaline (soda lye) solutions and does not always allow selective precipitation of the other metals contained in the processed steel dust [22,23,24].

On an industrial scale, the EZINEX process is currently used, where steelmaking dust is leached using an ammonia solution. The solution is then purified by cementation to produce a Pb-rich concentrate. Zinc is recovered from the solution in the form of cathodes by electrolysis. A modification of this process is the high-temperature pre-treatment of the zinc-bearing material, as a result of which material similar in composition to crude zinc oxide is sent for leaching. Such an integrated technology is called ENDUTEC/EZINEX [22,25]. The modified ZINCEX process involves leaching of steelmaking dust with a sulphuric acid solution at 40 °C. After the leaching process, the solution is purified and extracted, and zinc is obtained from the solution by electrolysis [26]. There is also a method involving the selective chlorination and evaporation of metals including zinc from steelmaking dust, whereby the source of chlorine can be pure Cl, hydrochloric acid and even chlorine-containing plastic waste. Currently, this method is considered for the selective recovery of metals from blast furnace slurries [27,28].

A literature analysis of EAF dust recycling processes allows us to conclude that there is a lack of technology for their comprehensive processing and full management of the resulting products and waste. To meet these problems, an ecological and sustainable comprehensive technology for the processing of zinc-bearing waste was developed [29]. Metallurgical waste in the form of steelmaking dust is 100% processed into products that can be economically utilised. The technology is much more efficient than the technologies used to date on an industrial scale to recycle steelmaking dust. The process is implemented as waste-free and the products are zinc oxide, iron alloys and slag aggregate. All these materials have a wide range of applications and physico-chemical parameters that guarantee a high market potential and commercial value. This paper will only present a part of the research on the remelting of EAF dust in an electric arc-resistance furnace.

## 2. Research Methodology

The starting material for the study was steelmaking dust with the chemical composition shown in Table 1.

**Figure 1:**
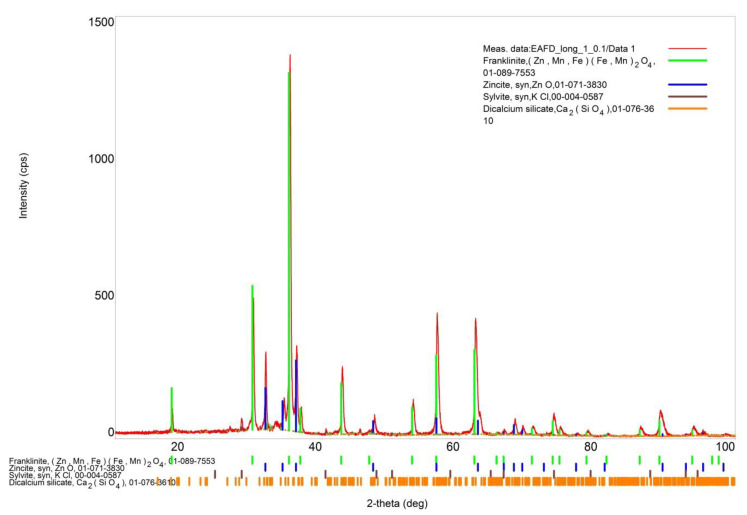
XRD analysis of steelmaking dust.

XRD phase analysis of the dust (Figure 1) indicates that franklinite and zinc oxide are the main phases. In addition, small amounts of potassium chloride and calcium silicate are present.

A sieve analysis was also carried out to determine the proportion of individual grain fractions in the metallurgical dust studied. The 0.4–0.63 mm fraction dominates in the material studied. Its share is 39%. It should be noted that over 80% of dust is within the granulation class of 0.2–1.0 mm.

Steelmaking dust is the waste material which should not be stored due to the leachability of certain components. Leachability was checked for this material according to TCLP [30,31]. It appears that leachability was exceeded for zinc, lead and cadmium.

The steelmaking dust was granulated with the addition of a reductor, which was coal dust containing more than 86% carbon. Steel dust mixtures with a reductor 15–25% were averaged in a mixer and then fed with the addition of water (10%) to a disc granulator. The granulator plate, 1.5 m in diameter and 245 mm high, was inclined at an angle of 45°. The rotational speed of the platter was 12 rpm. The feeding speed of the material to the granulator plate and the addition of water for granulating were fitted to obtain granules with a diameter of 8 to 15 mm. The resulting granules were dried in a rotary kiln to a moisture content of less than 0.1%. Granules with a fraction above 5 mm of more than 80% were obtained.

**Figure 2:**
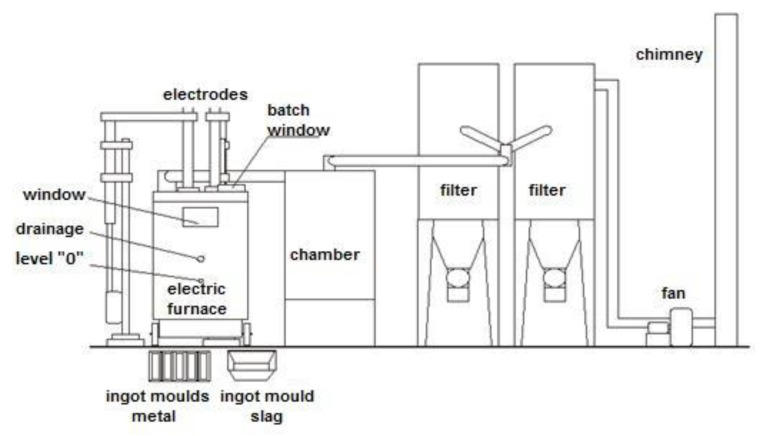
Diagram of the experimental electric furnace installation.

Melt tests on granulated steelmaking dust with reductor were carried out in the installation shown schematically in Figure 2, consisting of the following main components:

- Electric arc-resistance furnace with a core area of 0.16 m^2^;
- Afterburning chamber, which is a horizontal section of the gas extraction pipeline behind the furnace;
- Pre-dusting chamber and gas pre-cooling chamber;
- Fabric filter;
- Fan.

The procedure for carrying out granulate reduction in the electric furnace included:

- Periodic batching (4 times, every 20 min) and solid reduction of 200 kg of granules at 1250–1300 °C and a time of about 4 h;
- Reduction of liquid slag at 1400–1500 °C for 1.0–2.0 h;
- Drainage of metallic iron and slag at 1450–1550 °C into flat ingot moulds lined with refractory concrete with a zero opening;
- Zinc vapour combustion and CO afterburning in the furnace gas space, extraction pipeline and afterburning chamber by free atmospheric air intake through slots;
- Cooling of the process gases and pre-dusting in a cyclone cooler;
- De-dusting of the process gases in a bag filter.

During the solid pellet reduction, the electrodes were immersed in liquid slag under the solid charge layer. As the metal level rose, they were lifted upwards to avoid short-circuiting the current on the metal. By adjusting the voltage on the electrodes, the furnace output was as high as possible in the range of 50–60 kW.

After slag melting, reduction was carried out at an average furnace power in the range of 50–85 kW.

After completion of the solid granule reduction and after liquid slag reduction, a sample of slag and metal was taken from the furnace and their chemical composition was analysed with a handheld spectrometer for semi-quantitative coarse analyses.

The final products (slag and metal), after being knocked out of the ingot moulds, were separated and weighed. Product samples were then taken for chemical analysis.

Dust from the filter during each smelt was collected every 1 h of the process, and a weighed and an averaged sample was taken for analysis. At the end of each smelt, the dust removal system was cleaned of dust and the dust was weighed.

A number of process parameters were recorded during remelting:

- Time of each operation;
- The temperature of the products;
- Slag during solid reduction (lance with Pt-RhPt thermocouple);
- Slag before final discharge (optical pyrometer and lance with Pt-RhPt thermocouple);
- Slag and metal at the discharge chute with an optical pyrometer;
- Gas temperature;
- In the afterburning chamber;
- Behind the cooling chamber;
- Before the bag filter;
- Electricity consumption meter status at the beginning, at the beginning of each batch charging, after slag reduction, and after slag and metal discharge;
- Current, voltage at electrodes, power on secondary side;
- Consumption of electrodes by weighing the new electrodes inserted and the waste after cutting off the consumed electrodes;
- Continuous measurement of the composition of the gases in the afterburning chamber with the Siemens apparatus: CO_2_, O_2_, CO, SO_2_, H_2_;
- Continuous measurement of the gas composition before the bag filter: CO_2_, O_2_, CO, SO_2_, NO_2_;
- Periodic measurement of gas flow;
- Periodic measurement of dust content in gases in the melting phase (charging and reduction in the solid state) and in the liquid slag reduction phase.

## 3. Tests Results and Discussion

Three smelts of steel dust were made at reductor contents of 15 and 20%, and one each for 17.5 and 25% reductor. The smelting parameters are shown in Table 2.

**Figure 3:**
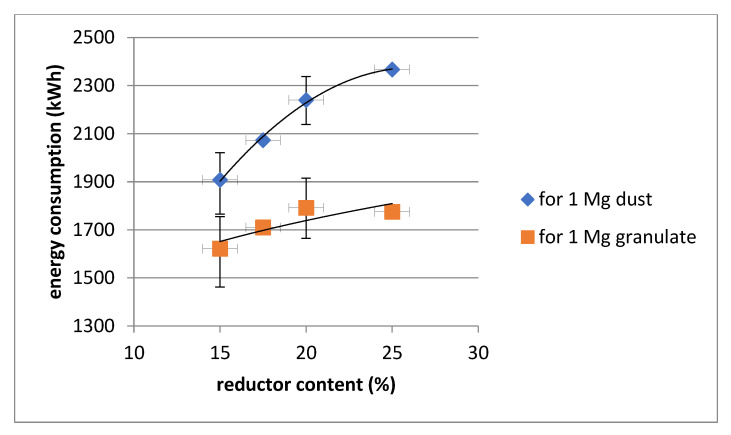
Energy consumption for smelting 1 Mg of dust and 1 Mg of granulate.

The smelting time was in the range of 5 to 6 h with exceptions of emergency situations (e.g., the need to service the electrodes). Energy consumption was determined for solid reduction and slag reduction in the liquid state. The energy consumption for slag reduction represented on average about 30% of the total energy consumption. However, it should be noted that the reduction of both zinc and iron occurred to a significant extent while still in the solid phase. However, additional energy consumption from the smelting of the charge was needed to increase the reduction of iron and to raise the temperature of the slag and smelt for better separation. The energy consumption was converted to the unit mass of steelmaking dust and granulate. An amount of 1 Mg of granules contained from 750 to 850 kg of steelmaking dust. Hence, after conversion, the energy consumption per 1 Mg of dust is from 1.18 to 1.33 times greater than that of granules. These dependencies as a function of the reductor share are shown in Figure 3. For reductor shares of 15 and 20%, the values were averaged.

It should be noted that energy consumption will always depend to some extent on the process time and temperature. However, in the smelts carried out, an attempt was made to maintain a uniform course of the process and its time depended on the achievement of certain temperatures. An attempt can therefore be made to determine the effect of the reductor on energy consumption. The resulting dependencies show that it is not advisable to increase the proportion of reductor as this increases the energy consumption. However, the process should continue until there is a successful conversion of zinc to dust and iron to alloy. This can be assessed on the basis of analyses of the smelt products, which are presented in Table 3, Table 4 and Table 5.

The chemical composition of filter dust shows high stability. High zinc content in the range of 62–69% and low lead content (2.5–3%) allow a correct assessment of its quality. The potassium and chlorine contents of 3.3% and 4.2%, respectively, are an undesirable feature. A variable manganese content of 0.57 to 2.82% results in a colour change of the dust from light yellow to grey-brown.

**Figure 4:**
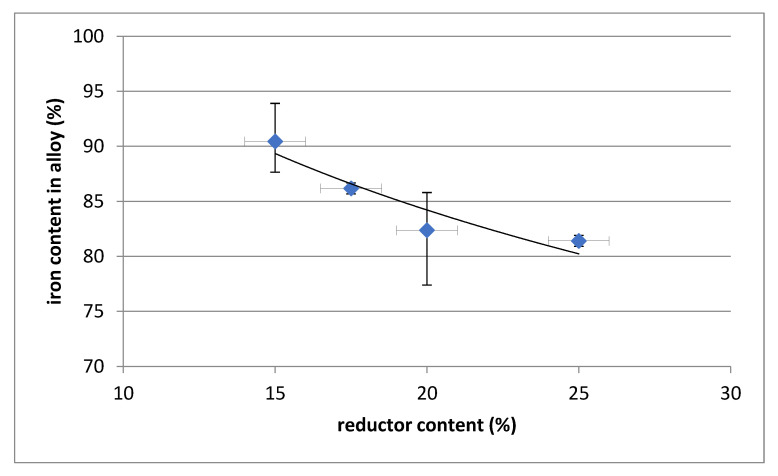
Dependence of iron content in the alloy on the content of reductor.

**Figure 5:**
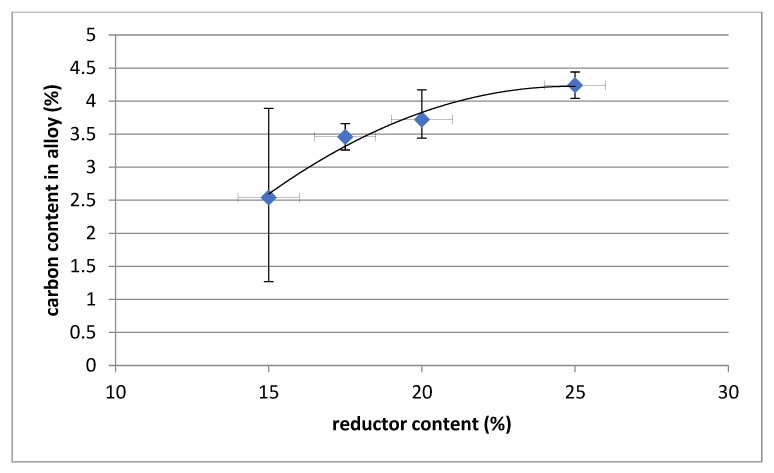
Dependence of the carbon content in the alloy on the content of reductor.

**Figure 6:**
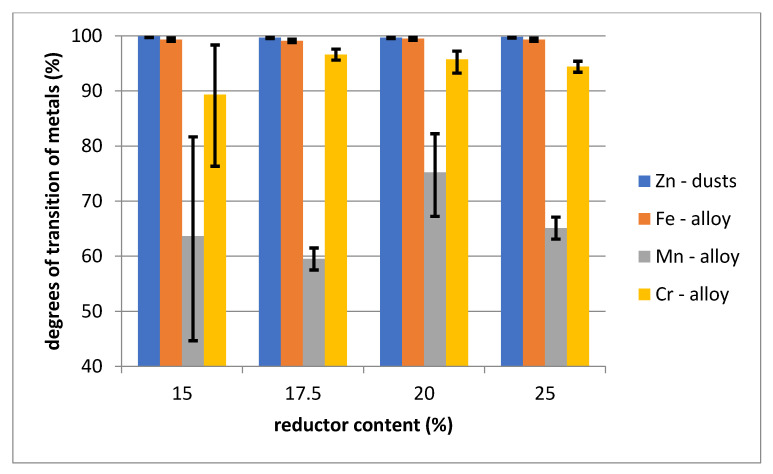
Dependence of the degrees of transition of metals to dust and alloy on the content of reductor.

The chemical composition of the polymetallic alloy indicates a high iron content (practically above 80%) and the presence of alloying steel components such as Mn, Cr and Si. The content of copper is at a level of approximately 0.4%. Due to the reductor used, the alloy generally contains more than 3% carbon. Higher reductor content increases the reduction of manganese, chromium and silicon with a corresponding decrease in iron content. A higher reductor content also results in an increase in the carbon content of the alloy. The dependency is shown in Figure 4, Figure 5 and Figure 6.

The slag obtained in smelting has a low content of zinc, lead and copper. The total content of these metals does not exceed 0.2%. The iron content is also low and, with one exception, does not exceed 1%. This indicates high recovery rates of zinc to dust and iron to alloy. This slag composition allows its use as an aggregate.

The use of waste slag as an aggregate may cause leaching of environmentally hazardous components. A leaching test was therefore carried out according to the TCLP (Toxicity Characteristic Leaching Procedure). Leaching was analysed by ICP OES method and the concentration of eluted elements in mg/L was obtained. Verification of the results obtained for the TCLP standard consisted only of direct comparison of the numerical values The obtained results are presented in Table 6.

Analysis of the leaching rate of slag components indicates very little possibility of the components passing into the leachate. In all cases the metal concentrations in the leachate are well below the permissible limits. This confirms the possibility of using the slag as an aggregate.

Significant elements of the conducted studies are the balances made on the basis of mass data and results of chemical analyses of the batch and products. The most interesting are the mass balance and the balances for zinc and iron. Performing precise melt balances is somewhat difficult. This is due to the escape of gases within the electrodes and through leaks in the dedusting system. This causes difficulties in the zinc–dust balance. Another problem is the estimation of the alloy weight caused by the formation of metal spatters during casting. Due to the aforementioned problems, a decision was made to carry out the balance in the following way:

- The zinc balance was made based on the contents of this metal in the charge and in the alloy and slag. Since only low content of this metal in the smelt and slag was obtained, the error in determining the amount of zinc in these phases is very small. Assuming that the remaining zinc goes to dust, a zinc transition rate of 99.3% was obtained from the charge to ZnO dust;
- The iron balance was made based on the content of this metal in the batch and in dust and slag. Their low content in the listed smelting products does not significantly affect the error in determining the amount of iron in the alloy. The transfer of iron into the alloy calculated on this basis is 98.5%;
- Based on the above calculations obtained from 1 Mg of steelmaking dust, the average amounts of the individual products will be: 360 kg ZnO dust, 400 kg alloy and 120 kg slag.

Attention should be paid to other determined process parameters. The temperature of the gases behind the furnace measured in the afterburning chamber during smelting increased from approximately 200 °C and reached a maximum of 617–1028 °C usually in half the time of smelt. The gases, after cooling in the cooler, had an average temperature for the smelt in the range 210–230 °C, reaching a maximum in the range 260–380 °C. After the air was sucked into the pipeline, the average gas temperature before the filter in the smelting section was 90–130 °C, reaching a maximum within the range of 160 °C.

Gas vacuum was maintained at the following levels: after the cooler—500 Pa, before the filters—1500 Pa and after the filters—2500 Pa. The pressure drop across the filters (filter resistance) was in the range 600–1200 Pa.

The average composition of gases in the afterburning chamber in the smelting cross-section was in the range 16.8–19.2% O_2_, 1.69–5.74% CO_2_, 0.026–0.21% CO and 0–0.013% SO_2_. The very low content of SO_2_ in the gases in the afterburning chamber indicates that it is bound in the dust already at the gas afterburning stage. The maximum values of CO and CO_2_ usually occurred when the gas-dust phase was pushed out of the furnace during batching and the furnace outlet opening and inlet to the cooling chamber were clogged with dust or the gas extraction by the fan was too low.

The average composition of the gases before the filter in the melt cross-section ranged from 19.0–19.9% O_2_, 1.09–2.17% CO_2_, 0.003–0.034% CO, 0–30.3 vpm NO and 0–3.7 vpm SO_2_. The very low concentration of SO_2_ in the gases means virtually no emission of this pollutant to the atmosphere due to its binding in the dust as sulphates.

The dust content in the gas before the filter during the reduction of steel dust granulate in the solid state (so-called smelting phase) was within the range of 7.85–18.8 g/m^3^N with an average value of 12.7 g/m^3^N. The dust content under normal conditions in the gas before the filter during the liquid slag reduction (so-called reduction phase) was in the range of 2.90–13.4 g/m^3^N averaging 7.12 g/m^3^N. A high dust content in gas at the liquid slag reduction stage, despite the fact that it already contains a very small amount of zinc, is a result of reduction by gases (CO) of dust and zinc oxide sinters deposited on the lining of the gas space of the furnace as well as lifting of dust deposited in pipelines.

Tests conducted on a semi-technical scale indicate high efficiency of smelting of steelmaking dust in the electric arc-resistance furnace. This applies particularly to the yields of zinc to dust and iron to alloy. It is very important that the slag obtained contains very small amounts of heavy metals and can be used as aggregate. The proposed process can therefore be described as waste-free. Despite slightly higher processing costs than the most popular Waelz technology, it does not generate slag with relatively high concentrations of zinc and lead. It also allows recovery of iron in the form of an alloy suitable for further processing. It should also be mentioned that the reductor can also be waste carbonaceous materials with high carbon content.

## 4. Conclusions

Steelmaking dust is a valuable source of metals, particularly zinc and iron. Most of the dust is processed using the Waelz process. This process makes it possible to recover zinc and lead in the form of dust. However, solid slags still contain relatively high levels of these metals. Additionally, virtually all of the iron passes into the slag. Disposal of such slag is difficult and not always carried out. The processing method proposed in this paper, tested on a semi-technical scale, does not have such disadvantages.

The results of the research carried out allow the following conclusions to be formulated:

- The most favourable effects of the processing of steelmaking dust in the arc-resistance furnace were obtained with the use of a reducer in the amount of 15% in relation to the weight of dust. This is particularly evident in the specific energy consumption, which averaged just over 1600 kWh/Mg batch.
- A high zinc to dust phase transition (ZnO) of 99.3% was achieved. The dust represents a product with high market value.
- A high degree of iron transition to metallic alloy of 98.5% was obtained. The alloy can be easily refined to steel.
- Slags with a low total Zn + Pb + Cu content of less than 0.2% were obtained. The results from the leaching test indicate that the slag is environmentally friendly. The slag can be used as an aggregate.
- The proposed processing of steelmaking dust does not generate condensed waste.

## Figures and Tables

**Table 1:** Chemical composition of steelmaking dust.

Element	Fe	Zn	Pb	Mn	Si	Cu	Cr	Ca	Cl
wt.%	36.69	23.95	1.24	4.44	1.74	0.18	0.30	4.35	0.87

**Table 2:** Smelting parameters.

Parameters	Unit	Smelt 1	Smelt 2	Smelt 3	Smelt 4	Smelt 5	Smelt 6	Smelt 7	Smelt 8
reductor content	%	15	15	15	17.5	20	20	20	25
total smelting time	h:min	05:05	05:30	06:05	05:35	06:40	05:40	06:10	05:50
solid state reduction time	h:min	03:55	04:00	04:00	04:00	05:10	04:00	04:00	04:00
slag reduction time	h:min	01:10	01:30	02:05	01:35	01:30	01:40	02:10	01:50
average temp. of the reduction in solid state	°C	1268	n.a.	1266	1314	1233	1295	1302	1300
average temperature of slag reduction	°C	n.a.	1412	1510	1422	1396	1525	1490	1434
slag tapping temperature	°C	1458	1500	1537	1467	1458	1480	1659	1553
energy consumption for solid state reduction	kWh	214	217	243	242	251	241	215	250
energy consumption for slag reduction	kWh	82	113	104	100	127	97	144	105
smelting energy consumption	kWh	296	330	347	342	378	338	359	355
energy consumption per 1 Mg of granules	kWh	1480	1650	1735	1710	1890	1690	1795	1775
energy consumption per 1 Mg of steel dust	kWh	1741	1941	2041	2073	2363	2113	2244	2367

**Table 3:** Chemical analysis of bag filter dust.

Content wt.%	Smelt 1	Smelt 2	Smelt 3	Smelt 4	Smelt 5	Smelt 6	Smelt 7	Smelt 8
Zn	61.8	66.6	66.6	63.2	57.1	69.3	67.8	62.6
Pb	2.56	2.63	2.46	2.72	3.00	2.50	2.49	2.43
Fe	0.29	0.23	0.19	0.36	0.46	0.09	0.11	0.35
Mn	2.82	0.78	0.89	1.89	2.10	0.57	0.80	2.30
K	3.41	3.31	3.41	3.48	3.40	3.34	3.33	3.24
Na	<0.01	<0.01	<0.01	<0.01	0.55	<0.01	<0.01	<0.01
Cl	4.02	4.23	4.48	4.19	4.24	4.33	4.44	4.02
F	0.18	0.19	0.19	0.20	0.18	0.15	0.18	0.19
S	0.75	0.58	0.78	0.74	1.03	0.66	0.59	0.81

**Table 4:** Chemical analysis of the metallic alloy.

Content wt.%	Smelt 1	Smelt 2	Smelt 3	Smelt 4	Smelt 5	Smelt 6	Smelt 7	Smelt 8
Fe	87.65	93.9	89.8	77.0	85.8	77.4	83.9	81.4
Mn	5.66	2.06	1.34	6.96	6.90	8.22	6.78	8.37
Cr	1.16	0.71	0.60	0.54	0.78	0.69	0.85	0.86
C	3.89	1.27	2.46	4.22	4.17	3.55	3.44	4.24
Si	0.28	0.34	1.41	4.66	0.71	1.88	0.93	1.46
Cu	0.43	0.45	0.48	0.38	0.38	0.40	0.35	0.31
Zn	0.03	0.02	0.25	0.17	0.11	0.21	0.25	0.13

**Table 5:** Chemical analysis of slag.

Content wt.%	Smelt 1	Smelt 2	Smelt 3	Smelt 4	Smelt 5	Smelt 6	Smelt 7	Smelt 8
Zn	0.04	0.02	0.02	0.01	0.01	0.02	0.01	0.02
Pb	0.067	0.0025	<0.0025	<0.0025	0.0026	0.0041	<0.0025	<0.0025
Cu	0.013	0.0081	0.0099	0.0061	0.0072	0.0078	0.0082	0.0085
Fe	0.59	0.55	1.86	0.45	0.60	0.92	0.64	0.70
Mn	5.05	6.00	13.1	5.49	1.30	7.30	6.05	5.49
Ca	16.2	16.4	16.6	18.2	17.4	16.4	17.0	18.7
Mg	6.94	6.96	5.12	5.98	6.51	7.30	6.87	5.92
Al	4.49	4.02	2.88	4.34	7.26	3.49	3.67	3.99
Si	15.4	15.0	12.6	15.4	15.5	14.2	14.9	15.3

**Table 6:** Concentration of elements in the leachate compared to the TCLP standard.

Element	Concentration (mg/L)	Max. Concentration (mg/L)
Zn	0.093	250
As	<0.1	5
Ba	1.74	100
Cd	<0.1	1
Cr	0.029	5
Pb	0.651	5
Hg	-	0.2
Se	-	1
Ag	0.012	5

## Data Availability

The data presented in this study are available on request from the corresponding author. The data are not publicly available due to the extremely large size.
